# Evaluating the Impact of Continuous Glucose Monitoring on Erectile Dysfunction in Type 1 Diabetes: A Focus on Reducing Glucose Variability and Inflammation

**DOI:** 10.3390/healthcare12181823

**Published:** 2024-09-12

**Authors:** Nicola Tecce, Davide Menafra, Mattia Proganò, Mario Felice Tecce, Rosario Pivonello, Annamaria Colao

**Affiliations:** 1 Department of Clinical Medicine and Surgery, Department of Endocrinology, University Federico II of Naples, 80138 Naples, Italy; davide.menafra@unina.it (D.M.); mattia.progano@outlook.it (M.P.); rosario.pivonello@unina.it (R.P.); amcolao58@gmail.com (A.C.); 2 Department of Pharmacy, University of Salerno, Via Giovanni Paolo II 132, 84084 Fisciano, Italy; tecce@unisa.it; 3 UNESCO Chair for Health Education and Sustainable Development, University Federico II of Naples, 80138 Naples, Italy

**Keywords:** type 1 diabetes, erectile dysfunction, continuous glucose monitoring, autonomic neuropathy, glucose variability, endothelial dysfunction, sexual health, quality of life, ED management

## Abstract

Type 1 diabetes (T1D) severely impairs metabolic control and can lead to erectile dysfunction (ED) through hyperglycemia-induced vascular damage, autonomic neuropathy, and psychological distress. This review examines the role of continuous glucose monitoring (CGM) in ameliorating ED by addressing glucose variability and inflammation. A comprehensive analysis of studies and clinical trials was conducted to evaluate the impact of CGM on metabolic control, inflammatory responses, and vascular health in patients with T1D. Evidence suggests that CGM systems significantly stabilize blood glucose levels and reduce hyper- and hypoglycemic episodes that contribute to endothelial dysfunction and ED. CGM’s real-time feedback helps patients optimize metabolic control, improve vascular health, and reduce inflammation. CGM has the potential to redefine ED management in patients with T1D by improving glycemic control and reducing the physiological stressors that cause ED, potentially improving quality of life and sexual health. Further research is warranted to explore the specific benefits of CGM for ED management.

## 1. Introduction

Type 1 diabetes (T1D), a chronic autoimmune disease characterized by the destruction of insulin-producing beta cells, poses significant health challenges beyond its immediate metabolic effects [1,2]. One troubling complication is erectile dysfunction (ED), with an estimated prevalence of 42% in men with T1D [3]. This risk is more than seven times higher compared to healthy individuals, stemming from a complex interplay of hyperglycemia-induced vascular damage, autonomic neuropathy, and psychological stress [3,4]. The pathophysiology involves endothelial dysfunction, reduced nitric oxide (NO) synthesis, oxidative stress, and advanced glycation end-products (AGEs), contributing to impaired vascular function and nerve damage [5,6]. Chronic hyperglycemia and glucose variability drive these mechanisms, exacerbating cardiovascular complications, through an increased and chronic inflammatory response [7,8,9,10,11]. These cardiovascular problems are exacerbated by the episodes of hypoglycemia seen in T1D, which have been independently associated with an increased risk of cardiovascular events [12,13,14]. In addition, the typical onset of T1D in childhood or adolescence means that the burden of T1D can accumulate over a longer period of time compared to people with type 2 diabetes, who typically develop the disease later in life [15,16,17]. In addition, early onset of diabetes, defined as onset within 10 years of age, has been shown to be an independent cardiovascular risk factor [18]. Advances in diabetes management, such as continuous glucose monitoring (CGM) systems, offer new opportunities for improving glycemic control and reducing the risk of diabetes-related complications, including ED. This review explores the potential benefits of CGM, particularly focusing on its ability to reduce glucose variability and inflammation, two critical factors implicated in the pathogenesis of ED in diabetes [5,19,20,21]. We will evaluate the impact of CGM on metabolic control, inflammatory responses, and vascular health and discuss how this technology could redefine the management strategies for ED in patients with T1D, potentially improving their quality of life and sexual health.

## 2. Aim

The primary aim of this review is to comprehensively examine the multifaceted relationship between glycemic control and the prevalence and severity of ED in patients with T1D. This review will explore both the organic and psychological impacts of glucose variability on erectile function, with a particular emphasis on the interplay between vascular health and nerve function. Additionally, this review aims to clarify the potential role of continuous glucose monitoring (CGM) systems in improving T1D-related ED by:

1. *Reducing Glucose Variability*: Evaluating how CGM helps stabilize blood glucose levels and reduce fluctuations, which are critical in preventing inflammation, endothelial dysfunction, and nerve damage associated with ED.
2. *Mitigating Inflammatory Responses*: Investigating the role of CGM in lowering markers of systemic inflammation, such as pro-inflammatory cytokines (e.g., TNF-α, IL-6), which are known to contribute to oxidative stress and vascular damage.
3. *Enhancing Vascular and Nerve Health*: Analyzing how improved glycemic control through CGM contributes to better vascular health, including increased nitric oxide (NO) availability and reduced oxidative stress, thereby supporting healthy erectile function.
4. *Proposing Future Research Directions*: Identifying gaps in current research and proposing future studies that could further elucidate the specific mechanisms through which CGM affects ED in T1D patients, including long-term outcomes and quality of life improvements.

## 3. Methods

**Figure 1:**
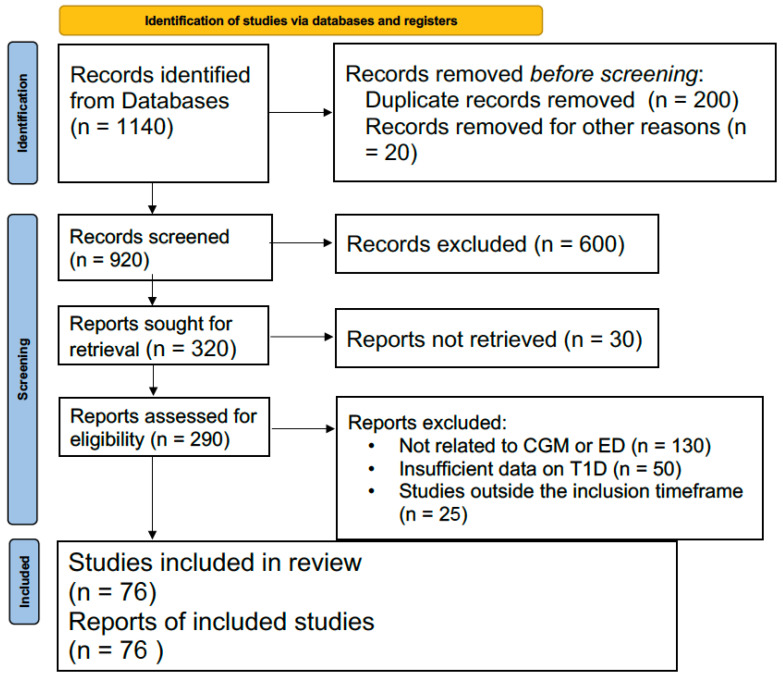
Methodology.

As shown in Figure 1, the review was conducted to assess the role of continuous glucose monitoring (CGM) in managing ED in patients with T1D. The literature search was performed across several databases, including PubMed, Scopus, and MEDLINE, as well as relevant clinical trial registers. The search strategy involved the use of specific keywords such as “Continuous Glucose Monitoring,” “Type 1 Diabetes,” “Erectile Dysfunction,” and “Inflammation,” targeting studies published within 2000 and 2024. A total of 1100 records were initially identified from databases and 40 records from trial registers. Following the removal of 200 duplicate records, 920 unique records were screened based on titles and abstracts. Manual screening was performed, as no automation tools were used in the exclusion process. A total of 600 records were excluded at this stage due to irrelevance to the study’s focus on CGM and ED. Following the screening, 320 reports were sought for full-text retrieval, but 30 reports were not retrieved. Full-text assessment was completed for 290 reports, and 210 were excluded for reasons such as lack of relevance to CGM or ED (n = 130), insufficient data on T1D (n = 50), and studies outside the inclusion timeframe (n = 25). Finally, 76 studies were included in the review. These studies were thoroughly evaluated for their methodological quality and relevance to the research question, with all included studies contributing to the final analysis of CGM’s potential role in reducing glucose variability and improving outcomes related to ED in T1D patients.

## 4. Biological Mechanisms Linking Type 1 Diabetes with Erectile Dysfunction

### 4.1. The Linkage between Dysglycemia and Inflammation in Type 1 Diabetes

**Figure 2:**
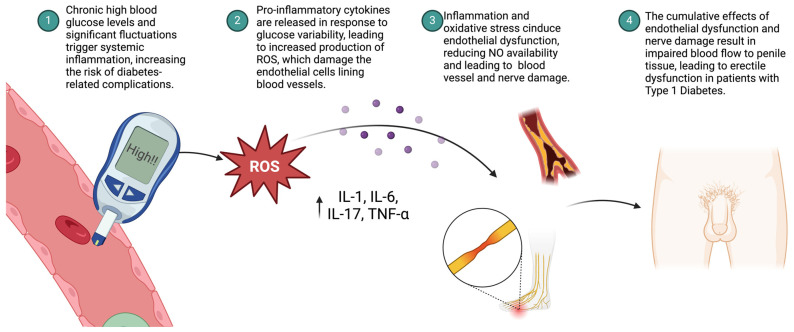
Link between inflammation and erectile disfunction in type 1 diabetes.

Dysglycemia, defined as chronic hyperglycemia and significant glucose variability, is a pivotal factor in the onset and progression of inflammation in individuals with T1D, as shown in Figure 2. Research by Matuschik et al. (2022) highlights how hyperglycemia exacerbates inflammation by reducing CD163 expression and increasing the release of proinflammatory cytokines in macrophages during hemoglobin-haptoglobin complex scavenging [6]. This study underscores the critical importance of glycemic control in preventing diabetic vascular complications and suggests potential therapeutic targets to mitigate these effects [6]. Similarly, Esposito et al. (2002) investigate the acute effects of hyperglycemia on inflammatory cytokine levels in humans, emphasizing the role of oxidative stress [22]. Their study, which utilized a hyperglycemic clamp in subjects with normal and impaired glucose tolerance (IGT), demonstrates that acute hyperglycemia significantly elevates circulating levels of proinflammatory cytokines such as IL-6, TNF-α, and IL-18 [22], as shown in Figure 2. Importantly, the administration of the antioxidant glutathione was shown to prevent this cytokine surge, highlighting the central role of oxidative stress in the inflammatory response triggered by hyperglycemia [22]. The persistent hyperglycemia of T1D results in the formation of advanced glycation end products (AGEs), which are produced when glucose molecules bind to proteins or lipids in the body [23]. AGEs then interact with their receptor, the receptor for advanced glycation end products (RAGE), initiating a cascade of pro-inflammatory signaling pathways [23]. The interaction between AGEs and RAGE activates immune cells, leading to the release of pro-inflammatory cytokines, including TNF-α and IL-6, which further exacerbate the inflammatory state [23]. This chronic inflammation not only responds to the hyperglycemic environment but also contributes to the deterioration of vascular health, significantly influencing the development of complications such as ED in T1D patients [23]. Further exploring this connection, Chang and Yang (2016) delve into the intricate relationship between hyperglycemia, tumorigenesis, and chronic inflammation [24]. Their study reveals how hyperglycemia, a hallmark of diabetes, fuels cancer progression by providing energy to malignant cells and promoting a pro-tumorigenic microenvironment through chronic inflammation [24]. This underscores the broader implications of managing hyperglycemia, not just for diabetes control but also for potentially reducing cancer risk, particularly in patients with chronic hyperglycemic conditions.

Moreover, as shown in Figure 2, dysglycemia-induced inflammation also impacts the nervous system, where an inflammatory environment can lead to nerve damage or neuropathy, further complicating the pathophysiology of ED [23,25]. Continuous glucose monitoring (CGM) holds the potential to reduce glucose variability, thereby lowering the production of AGEs and minimizing inflammation, which could be crucial in mitigating the progression of such complications in T1D [25]. This persistent hyperglycemia results in the formation of advanced glycation end products (AGEs), which are produced when glucose molecules bind to proteins or lipids within the body [25]. AGEs then interact with their receptor, designated as the receptor for advanced glycation end products (RAGE), thereby initiating a cascade of pro-inflammatory signaling pathways [23,25].

This interaction between AGEs and RAGE results in the activation of immune cells, which subsequently release pro-inflammatory cytokines, including TNF-α and IL-6 [23]. These cytokines serve to exacerbate the inflammatory state, thereby contributing to oxidative stress and further endothelial damage [23]. This chronic inflammation is not only a response to the hyperglycemic environment but also contributes to the deterioration of vascular health, which is a significant factor in the development of complications such as ED in patients with T1D.

### 4.2. Biological Mechanisms Linking T1D with ED

ED is defined as the inability to achieve or maintain an erection that is sufficient for satisfactory sexual performance and affects a significant proportion of men [26,27], particularly men affected by T1D and type 2 diabetes (T2D) [28].

**Figure 3:**
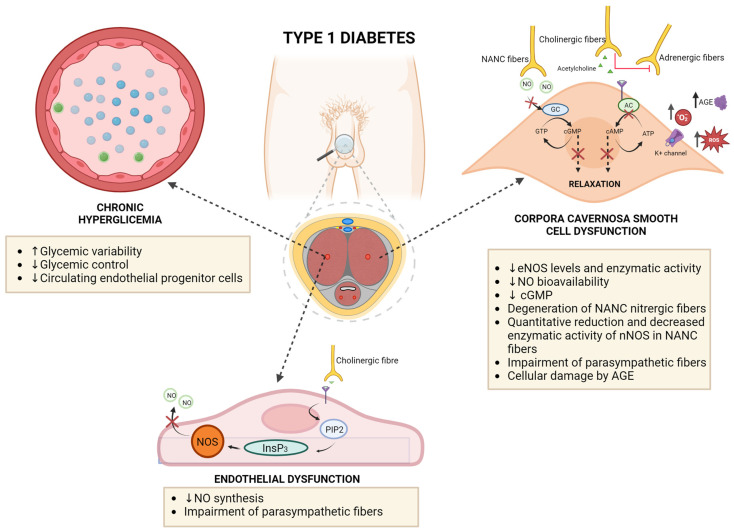
Biological mechanisms linking T1D with ED.

In physiological conditions, the passage of the penis from the flaccid state to the erect state is guaranteed by sexual stimulation, which activates the increasing of cGMP levels responsible for relaxation of smooth muscle cells in corpora cavernosa [29], as shown in Figure 3. The increased production of cGMP is induced by the local release of nitric oxide (NO) by the non-adrenergic non-cholinergic (NANC) fibers and the parasympathetic system, which, through the release of acetylcholine, activates the release of NO by the endothelial cells of the cavernous arteries, also stimulated by the shear stress deriving from the increased arterial inflow, with further amplification of the vasodilation process in the corpora cavernosa; moreover, the high intracorporal pressures characteristic of full erection are possible only with the concurrent limiting of the outflow of penile blood, induced by mechanical occlusion of venous outflow between the semi-rigid tunica albuginea and the expanding sinusoids [26,29].

As shown in Figure 3, the alterations of one or more of the mechanisms could contribute to the occurrence of ED. Several preclinical studies have demonstrated that in T1D the main mechanisms involved in the pathogenesis of ED are attributable to the alteration of the process of cavernous vasodilation [28,30,31]. Specifically, the presence of a significant degeneration of cavernous nitrergic fibers and parasympathetic nervous transmission has been demonstrated in experimental mouse models of T1D, with a contextually significant reduction in the enzymatic activity of neuronal Nitric Oxide Synthase (nNOS) [32]; furthermore, a significant reduction in the enzymatic activity of endothelial nitric oxide Synthase (eNOS) and a reduction in the bioavailability of NO have been demonstrated at the endothelial level and in corpora cavernosa, probably due to NO activity as an ROS scavenger with a consequent negative effect induced by the increased oxidative stress associated with diabetes [28,33].

By a clinical point of view, prevalence of ED in T1D was estimated in a range between 37.5 and 42.5% [3,34], and the risk of developing ED has been estimated to be more than 7 times greater in males affected by T1D compared to healthy ones [3].

Some clinical factors have been characterized as having a greater impact on the risk of ED associated with T1D; in particular, age, microvascular complications, duration of T1D, and HbA1c levels represent the factors most affecting the risk of ED in T1D [3]. In particular, the role of HbA1c, a well-known marker of glycemic compensation and the stability of glycemic levels in diabetes, is particularly impactful and evident, thus underlining the importance of controlling the pathology and reducing glycemic variability as important strategies for preventing and reducing the severity of ED in T1D [35]. In this context, the significant frequency and notable negative impact of ED in T1D are therefore evident, and it becomes essential to characterize this complication from a clinical, pathophysiological, and instrumental point of view in order to improve the prevention and treatment of ED in T1D.

### 4.3. Role of Endothelial Dysfunction, Diabetic Neuropathy and Glucose Variability in the Development of ED in T1D

Endothelial dysfunction represents the main mechanism leading to the onset of ED in T1D [36]. Notably, endothelial dysfunction has been associated with the presence of chronic hyperglycemia and has been identified as the pathogenetic link between ED and CVD. Specifically, endothelial dysfunction induced by T1D mainly results in a reduction in NO synthesis (Figure 3); indeed, a study on a mouse model of streptozocin-induced T1D showed eNOS and NO levels synthesized by endothelial cells were significantly reduced in the corpora cavernosa, with evidence of reversion of these alterations after eNOS gene transfer [37]. Moreover, in the same experimental mouse model of steptozocin-induced T1D, a significant reduction in NO bioavailability and consequently of cGMP levels in corpora cavernosa, induced by the scavenger effect of increased levels of ROS and superoxide anion, was demonstrated; otherwise, this molecular alteration has also been demonstrated to be reversed after superoxide dismutase gene transfer, determining an increased catabolism of ROS and superoxide anion, therefore contributing to the restoration of normal NO bioavailability and cGMP levels [38].

Furthermore, in a mouse model of streptozotocin-induced T1D, selective damage of cavernosal nitrergic fibers was demonstrated; in particular, a structural degeneration of NANC nitrergic nerves, a quantitative reduction, and a decreased enzymatic activity of nNOS in these fibers were reported in this experimental model, with the additional interesting evidence of a NO-dependent nature of this selective damage, probably dependent by the diabetes-related increased oxidative stress that induced the generation of potent free radicals with NO, such as peroxynitrite [39]. Consistently, a decreased cavernosal relaxation after electric field stimulation was demonstrated in experimental animals affected by T1D; nevertheless, a concomitant sympathetic antagonist was applied; this effect was hypothesized to be accountable to the aforementioned structural and functional damage of nitrergic nerves but also impairment of parasympathetic nerves and their related cholinergic stimulus physiologically responsible for smooth cell relaxation and endothelium-induced NO release [28]. In particular, diabetes-induced neurological damage has been demonstrated to have two phases in this experimental model: in the first phase, nitrergic nerve fibers lose some of their neuronal nitric oxide synthase content and function, and this phase seemed to be reversible; in the second phase, nitrergic degeneration takes place in the cell bodies in the ganglia, leading to complete loss of nitrergic function, and this phase is irreversible since it is due to neurodegeneration and apoptotic cell death in the ganglia [32].

Another important role in the induction of ED in T1D is played by AGEs (Figure 3): they are the products of non-enzymatic reactions between glucose and lipids, proteins, or nucleic acids and have been characterized as being significantly elevated in the corpora cavernosa in male patients with diabetes [5,40]; specifically, the molecular role of AGEs in the early induction of ED in diabetes appears to be attributable to damage to cavernous smooth muscle cells and their potassium channels involved in the facilitation of intracellular calcium release and then in smooth muscle cell relaxation [41]. Consistently, the existence of a specific role of AGEs in the induction of T1D-related ED has been demonstrated through the evidence that AGEs are more prevalent in penile tissue from diabetic than in control animals, and aminoguanidine, normally preventing AGE formation, reversed the impairment in neuronal and endothelial NO-mediated penile smooth muscle relaxation seen in diabetes [40].

Additionally, a specific pathophysiological role in T1D-related ED has been attributed to circulating endothelial progenitor cells (Figure 3); in particular, these cells have been demonstrated to be globally significantly reduced in patients affected by T1D and ED, and their number is significantly correlated with testosterone serum levels, therefore highlighting the potential additional contribution of diabetes-related hypogonadism in the reduction in circulating endothelial progenitor cells and then in endothelial dysfunction responsible for ED [42].

Furthermore, taking into account the clinical impact of ED in T1D and specific clinical factors contributing to the risk of ED, in a longitudinal observational study it was shown that the prevalence of ED in young males with T1D was 37%, which was superimposable based on what has already been reported in the literature; moreover, this study confirmed the role of certain T1D-specific factors in determining ED, including poorer glycemic control [43]. Consistently, a fundamental role in the etiopathogenesis of ED associated with T1D is certainly attributable to the adequacy of glycemic control as well as the variability of glycemic profiles. In this sense, a study on T1D patients included in the Diabetes Control and Complications Trial (DCCT) evaluated the impact of intensive glycemic control and reduction in glycemic variability on the risk of ED in these patients by dividing them into two subgroups, one with T1D for 5 years or less without microvascular complications and one with T1D for 15 years or less with microvascular complications; in particular, the results of this analysis demonstrated a lower prevalence of ED in patients of the first subgroup subjected to intensive glycemic control compared to those subjected to conventional treatment, highlighting a significant association in both subgroups between HbA1c values and risk of ED, and thus confirming the higher risk of ED related to poorer glycemic control and underlining the importance of intensive glycemic control and reduction in glycemic variability as strategies to reduce the risk of ED in patients with T1D [35]. According to this evidence, it appears as really important to highlight the wide emerging role attributed to glycemic variability as a clinical factor involved in the risk of diabetes complications; indeed, glycemic variability, defined as the degree to which a patient’s blood glucose level fluctuates between high (peaks) and low (nadir) levels, has been proposed and endorsed as a new diabetes control-related risk factor above HbA1c for vascular complications of diabetes [44].

Summarizing, ED represents a significant complication of T1D that should not be underestimated; the occurrence of ED in T1D could be explained by the integrated role of different pathophysiological elements, such as endothelial dysfunction and reduction in endothelial cell precursors, diabetic neuropathy and AGEs-mediated damages, and clinical factors, such as glycemic control and glycemic variability, which should all be taken into account when ED occurrence and etiopathogenesis are studied in male patients affected by T1D.

## 5. CGM Technology as Strategy to Reduce Glucose Variability and Inflammation: Is There a Role for the Improvement of ED in T1D Patients?

### 5.1. CGM Technologies in T1D: Types and Benefits

**Figure 4:**
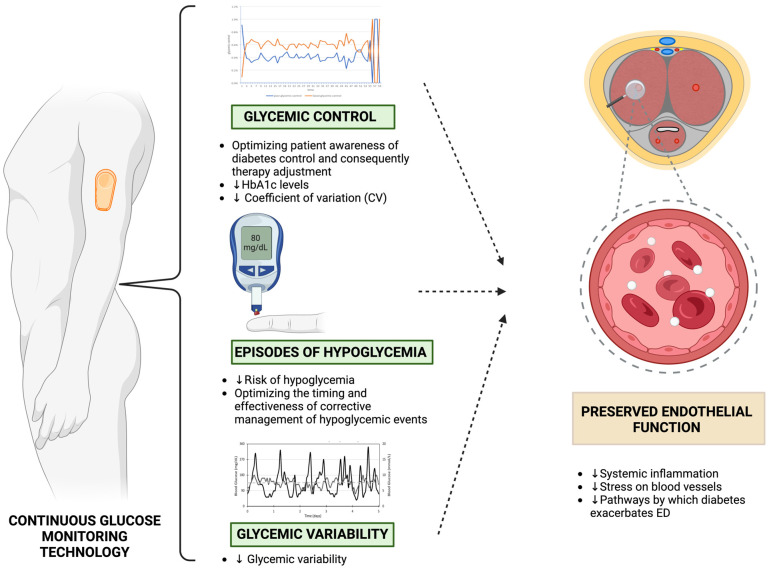
Impact of CGM in preventing endothelial damage.

Continuous glucose monitoring (CGM) systems provide a comprehensive overview of glucose levels, allowing individuals with T1D to manage their condition more effectively (Figure 4) [45,46,47]. CGMs use a small sensor that is placed under the skin to continuously measure glucose levels. This sensor is connected to a transmitter that sends data to a device such as a smartphone or insulin pump (Figure 4). There are two types of CGM: traditional, which sends glucose information automatically and continuously, and flash glucose monitoring (FGM), which requires the user to scan the sensor to retrieve glucose data. Both types track current and past glucose levels, trends, and predicted future levels. CGMs are divided into real-time and professional (retrospective) types. Personal CGMs provide people with diabetes (PWD) with immediate access to their glucose data, allowing for real-time intervention [48]. Professional CGMs, which are less common, keep the data hidden from the user and only accessible to clinicians after the fact [48]. These devices measure glucose concentrations in real time and continuously throughout the day, providing data that can alert users to glucose fluctuations [49]. This information is crucial for timely adjustments in diet or insulin therapy, significantly reducing the risk and severity of hypoglycemia and improving overall glucose management [49].

The benefits of CGM in managing T1D are well documented. Studies have shown that CGM systems can significantly improve glycemic control, primarily through reduced glucose variability [50,51]. This stabilization of glucose levels is particularly beneficial for preventing the fluctuations that can lead to both short-term complications, such as diabetic ketoacidosis, and long-term complications [52]. By offering a detailed view of glucose trends, CGM helps in fine-tuning insulin doses and dietary choices, which enhances the quality of life for patients by allowing more precise and less intrusive management of their condition.

Furthermore, evidence supporting the effectiveness of CGM highlights its ability to increase the time patients spend in their target glycemic range [53]. This not only reduces episodes of both hypo- and hyperglycemia but also contributes to better long-term health outcomes [54]. The continuous feedback provided by CGM enables users to understand their glucose patterns better and predict potential episodes before they become severe, offering a proactive approach to diabetes management [55].

### 5.2. Benefits of CGMs in Improving Glycemic Control in T1D

The use of CGM has been widely demonstrated to be an instrument contributing to the improvement of glycemic control in different forms of diabetes [56]. In particular, CGM has been reported to provide a significant advantage in the glycemic control in patients affected by T1D (Figure 4); in this context, CGM has been demonstrated to be able to induce a reduction of 0.64% of HbA1c in patients affected by T1D using CGM compared with patients using standard monitoring systems of blood glucose, specifically in patients using CGM for longer time, namely between 40 and 60% of the time, and with a double effect in patients using CGM for more than 80% of the time [56,57,58]. Consistently, another trial showed a reduction in HbA1c of 0.5% in patients affected by T1D using CGM for at least 6 days/week compared with patients using standard monitoring systems of blood glucose [58]. Overall, this evidence strongly supports the capability of CGM of improving glycemic control in T1D, mainly by optimizing the awareness of patients of diabetes control and consequently the therapy adjustment; the improvement of glycemic control is undoubtedly one crucial point of the strategies focusing on the reduction in T1D complications, and specifically the amelioration of glycemic control granted by the use of CGM in male patients affected by T1D could represent an instrument to reduce the risk and severity of ED in this setting of patients. Indeed, glycemic control expressed as HbA1c levels has been demonstrated to represent one of the most impactful factors in the reduction in the risk of ED in T1D male patients, since it has been demonstrated that intensive treatment, with consequently significantly lower HbA1c levels than those granted by conventional treatment, was associated with a reduced risk of ED in patients affected by T1D [35].

### 5.3. CGMs Reduces the Risk of Hypoglycemia in T1D

Moreover, the use of CGM in patients affected by T1D has been recognized to have the fundamental merit of significantly reducing the risk of hypoglycemia and optimizing the time and effectiveness of its corrective management mainly through the real-time availability of glycemic levels for patient (Figure 4) [56]; in particular, a multicenter clinical study, involving T1D patients characterized by a good baseline glycemic control, have demonstrated that the use of CGM systems granted a reduction of 50% of time spent in controlling hypoglycemic events, nevertheless a contextual amelioration of HbA1c was not demonstrated probably for the absence of a structured educational activity addressed to improve patients awareness and ability in diabetes management [59]; however, this evidence supported the concept of a significant reduction in hypoglycemic events in T1D patients promoted by the use of CGM, with consequent decrease of hypoglycemia-related adverse events and complications. It is well established that hypoglycemia, typically defined biochemically as a plasma glucose concentration of less than 63 mg/dL, is associated with a range of undesirable signs and symptoms [60]. These are mediated by the hyperactivation of the sympathetic nervous system, including sweating, shaking, and palpitations [60]. Furthermore, they can lead to a number of cardiovascular complications, both in the short and long term [60]. In the short term, these can include bradycardia and tachycardia, which carry an increased risk of severe arrhythmias and lowered potassium serum levels [60]. Severe arrhythmias and decreased serum potassium levels are driven by a hypoglycemia-induced adrenergic response and widened arterial pulse pressure [60]. Long-term complications include endothelial dysfunction, an inflammatory response, and a systemic coagulopathy, which impair overall cardiovascular health and subsequently erectile function [60]. Additionally, negative short-term and long-term psychological effects related to hypoglycemic events further impair erectile function [60].

### 5.4. CGMs and Glycemic Variability

Furthermore, CGM has been demonstrated to significantly contribute to the reduction in patients affected by T1D of glycemic variability (Figure 4), which has been recently described as a clinical factor impacting the risk of diabetes-related complications [56,60]. In particular, the use of CGM has been demonstrated to significantly reduce glycemic variability in cohorts of very young patients affected by T1D, resulting in a significant reduction in glucose standard deviation and range of glycemic excursions when compared to traditional glucose monitoring systems [20,56,61]. This evidence stressed the important role of CGM in reducing glycemic variability and its related complications; in this sense, ED has also been correlated with glycemic variability in patients affected by T1D. Specifically, obtaining a better glycemic control and a reduced glycemic variability has been demonstrated to significantly contribute to decreasing the risk of ED in T1D patients subjected to an intensive glycemic control compared to those subjected to conventional treatment [35], therefore suggesting that use of CGM in T1D and its ability to reduce glycemic variability could further contribute to reduce ED risk and severity in these patients. Conversely, another study evaluated the potential impact of glycemic variability on sexual dysfunctions, including ED, in male patients affected by T1D, comparing two subgroups characterized by high or low glycemic variability according to the value of the coefficient describing glycemic variability higher or lower than 36%; specifically, this study demonstrated the absence of significant differences between two subgroups, therefore suggesting a no clear role of glycemic variability in the etiology and risk of ED in T1D patients [62]. However, this apparent contradiction with the other evidence could be probably explained by the very young age of patients in the last described study, which implied a very short-term period of exposition for patients to glycemic variability, whereas this clinical factor has been described as involved in increased cardiovascular and ED risk in T1D when considered in a long-term period.

Schubert-Olesen O et al. explored the multifaceted benefits of continuous glucose monitoring (CGM) in enhancing physical activity regimens for individuals with type 2 diabetes [63]. By demonstrating how CGM can lead to improved glycemic control and minimize adverse events like hypoglycemia post-exercise, this review underpins the critical role of CGM in optimizing diabetes management through active lifestyles [63].

In summary, CGM experience provided a significant upgrade in the management of T1D and its complications, particularly its capability of improving glycemic control. Hypoglycemic events and glycemic variability could also significantly contribute to reducing the risk and severity of ED associated with T1D.

### 5.5. Comparison of CGM with Other Glucose Monitoring Technologies in the Improvement of Endothelial Dysfunction

Continuous glucose monitoring (CGM) systems have significant advantages over traditional glucose monitoring methods in managing endothelial function and reducing cardiovascular risk, particularly in patients with type 1 diabetes. Enhanced detection of glycemic variability is one of CGM’s key strengths. For example, a study demonstrated that real-time CGM significantly improved endothelial function as measured by brachial artery flow-mediated dilation in adolescents with type 1 diabetes [20]. CGM’s ability to monitor glucose levels continuously allows for the identification and mitigation of glycemic fluctuations, which are closely linked to endothelial health and cardiovascular risk [20].

Traditional glucose monitoring methods, which provide only snapshots of glucose levels, may overlook these critical fluctuations that impact the endothelium. CGM, however, offers continuous insight into glucose trends, enabling a more nuanced understanding and management of factors that influence endothelial function and the progression of atherosclerosis in diabetic patients.

In summary, CGM provides a detailed and dynamic view of glucose levels, crucial for safeguarding endothelial health and potentially mitigating long-term cardiovascular risks in diabetic patients. This continuous data collection is key in identifying patterns that might go unnoticed with intermittent testing, thus providing a substantial benefit in managing conditions that affect endothelial function.

### 5.6. Impact of CGMs on Inflammation and Sexual Health

Continuous glucose monitoring (CGM) plays a crucial role in mitigating ED by reducing glucose fluctuations and time spent in dysglycemia, both of which are closely linked to chronic inflammation and vascular dysfunction. Stabilizing blood glucose levels through CGM use decreases the production of reactive oxygen species (ROS) and inflammatory cytokines, which can damage the endothelial cells lining blood vessels [64]. Endothelial dysfunction results in reduced nitric NO availability, which is necessary for the dilation of blood vessels and the ability to achieve and maintain an erection [64]. Studies have demonstrated that poor glycemic control correlates with decreased NO levels, increasing the risk of vascular complications, including ED [64]. Additionally, inflammation caused by chronic hyperglycemia contributes to neuropathy, further increasing the risk of ED. By helping individuals with T1D maintain better glucose control, CGM reduces systemic inflammation, improves vascular health, and protects nerve function. The reduction in oxidative stress and inflammation facilitated by CGM use fosters healthier endothelial responses and improved blood flow, which can help prevent or alleviate ED in men with T1D [64].

However, limited data exist on the specific impact of CGM on sexual and erectile function in male T1D patients. However, some studies evaluating the use of CGM in diabetes could represent an interesting input to speculate on the possible impact of diabetes technology, in particular CGM use, in T1D both on quality of life and sexual health [65]. One study did not find a significant difference in sexual and erectile function between patients using modern technological approaches, like CGM, and those following traditional insulin therapy regimens [43]. In contrast, another study in T2DM patients found that using continuous subcutaneous insulin infusion (CSII) and CGM significantly reduced ED severity, supporting the idea that decreased glycemic variability could mitigate ED risk in T1D as well [66]. While the evidence for CGM’s role in reducing ED is promising, there is conflicting evidence about the specific effects of different insulin therapies like CSII and multiple daily injections (MDI) on ED. Additional studies are needed to clarify this, particularly focusing on T1D populations. Importantly, CGM and insulin pumps do not appear to have a significant negative impact on anxiety, body image, or sexual activity, with male patients sometimes disconnecting devices like CSII during intercourse to minimize discomfort [67]. These findings underscore the need for more discreet and less intrusive diabetes technologies that can improve both metabolic control and quality of life. Consistently, another study evaluated the impact of diabetes technology, particularly external devices such as CGM and CSII, demonstrating the lack of their detrimental effects on anxiety, body image, and sexual activity in people with T1D and the frequent adoption of the disconnection of external devices, particularly insulin pumps, during sexual intercourses in order to minimize the discomfort during sexual intimacy [68].

Overall, the evidence suggests that CGM may play a positive role in reducing ED risk and severity in men with T1D by lowering long-term glycemic variability. These findings may also encourage acceptance of CSII and CGM among patients who initially hesitate to use these technologies due to concerns about their impact on sexual health.

### 5.7. Limitations of Continuous Glucose Monitoring (CGM)

Although continuous glucose monitoring (CGM) systems have markedly enhanced diabetes management by furnishing real-time glucose data and curbing the dependence on fingerstick testing, they are not without technical constraints that can affect their efficacy and dependability.

A significant drawback of CGM systems is their accuracy, particularly during periods of rapid glucose fluctuations [69]. Continuous glucose monitors (CGMs) measure interstitial fluid glucose levels, which may lag behind blood glucose levels by 5 to 20 min [69]. This lag in measurement becomes problematic when glucose levels are rising or falling rapidly, such as in the postprandial period or during hypoglycemia [69]. Furthermore, some CGM devices necessitate periodic calibration with fingerstick blood glucose readings to preserve their accuracy, which can be onerous for users [69].

Furthermore, the placement of the sensor and the manner of its wear are also critical factors influencing the performance of CGM devices [70]. The sensors are typically positioned on the abdomen or upper arm and require replacement every 7 to 14 days [70]. The accuracy of the sensor may be compromised by a number of factors, including skin conditions, scarring, or the amount of subcutaneous fat at the insertion site [70]. This can result in potential inaccuracies or sensor failure. Moreover, the presence of certain substances, including vitamin C and acetaminophen, can impede the sensor’s readings, leading to the falsification of glucose levels [70]. This is a significant issue for users who frequently ingest these substances, necessitating supplementary monitoring to guarantee precise glucose management [70].

Furthermore, data transmission and connectivity present additional challenges [70]. CGM systems depend on wireless data transmission from the sensor to a receiver or smartphone, with a typical transmission range of approximately 20 feet [70]. In the event that the device is no longer within the designated transmission range, the connection may be terminated, resulting in the disruption of real-time monitoring and alerts [70]. This limitation can be inconvenient for users who rely on constant glucose monitoring, particularly during activities where they may be separated from their device [70].

The financial burden associated with CGM systems represents a substantial obstacle to their broader implementation [71]. Despite improvements in insurance coverage, the out-of-pocket costs for sensors, transmitters, and receivers remain considerable, particularly for individuals without comprehensive insurance coverage [71]. This financial burden represents a significant barrier to access for CGM technology, particularly among lower-income populations [71].

In clinical settings, particularly in hospitals, the use of CGM is also fraught with challenges [72]. The lag between interstitial and blood glucose readings is more pronounced in conditions such as hypoperfusion, hypotension, or during rapid changes in glucose levels, which renders CGM less reliable for critically ill patients [70,72]. Furthermore, integrating CGM data with electronic health record (EHR) systems can complicate patient monitoring and data accuracy during hospital stays [70,72].

These limitations underscore the necessity for continued advancement and enhancement of CGM technology to enhance its accuracy, reliability, and accessibility, thereby ensuring its effective utilization by all patients who could benefit from this sophisticated monitoring tool.

## 6. Future Research Directions for Continuous Glucose Monitoring (CGM) and Erectile Dysfunction in Type 1 Diabetes

Future research on continuous glucose monitoring (CGM) and ED in T1D should aim to address several underexplored areas. One significant gap is the long-term impact of CGM on ED. While the short-term benefits of CGM in controlling glucose variability are well documented, there is a need for longitudinal studies to evaluate its effects over several years. Such studies should track vascular and nerve health improvements and determine whether long-term CGM use can delay or reverse erectile dysfunction by consistently stabilizing glucose levels and reducing systemic inflammation. The integration of CGM in studies examining diabetic vascular complications remains essential to understanding its potential for mitigating long-term ED risks [70]. Moreover, further research is needed on how CGM affects inflammation, particularly the relationship between glycemic stability and reductions in inflammatory markers like TNF-α and IL-6, which play a role in the pathogenesis of ED. Since inflammation is a significant contributor to endothelial and nerve dysfunction in diabetes, future studies should focus on determining whether CGM’s ability to lower glucose variability directly translates into a measurable decrease in inflammation and oxidative stress. This area of investigation remains critical to evaluating CGM’s broader impact on diabetic complications, including ED.

In addition to its physiological effects, the psychological impact of CGM on sexual health also deserves further exploration [73,74]. CGM has been associated with reduced anxiety over glucose management, but whether this reduction in stress translates into improvements in sexual confidence or performance, particularly during intimate moments, has not been thoroughly studied. Future research could explore whether the psychological benefits of CGM contribute to improved sexual health outcomes in T1D patients, particularly in reducing anxiety-induced ED.

Finally, studies should also explore the gender-specific impacts of CGM. While ED has been a primary focus in men, the effects of CGM on sexual dysfunction in women with T1D have been largely overlooked [75]. Addressing gender differences in how CGM affects sexual health would provide a more comprehensive understanding of CGM’s impact on overall sexual well-being.

## 7. Discussion

The incorporation of continuous glucose monitoring into the management of T1D represents a significant advancement in the treatment of complications such as ED. CGM enables real-time glucose monitoring, allowing for precise insulin dosing and minimizing fluctuations that contribute to systemic inflammation and endothelial dysfunction, which are key factors in ED. By maintaining stable glucose levels, CGM reduces oxidative stress and the formation of AGEs, thereby preserving vascular health. Furthermore, this reduces the incidence of both hypoglycemia and prolonged hyperglycemia, which in turn lowers the inflammatory responses linked to endothelial damage and neuropathy. Furthermore, CGM alleviates the psychological burden associated with T1D management, which contributes to enhanced mental well-being and sexual health. Although existing evidence indicates that CGM may reduce the risk and severity of ED, further targeted research is required to substantiate this claim. Long-term studies should investigate the impact of CGM on glycemic variability, inflammation, and hypoglycemia to further validate its role in improving sexual health. The establishment of CGM-based protocols focused on ED will assist clinicians in addressing the underlying causes in a more efficacious manner.

## 8. Conclusions

Continuous glucose monitoring represents a pivotal advancement in the management of ED in men with type 1 diabetes mellitus. By stabilizing glucose levels and reducing both inflammatory and psychological stress, CGM addresses the physiological and mental components of ED. While the results are promising, further research is necessary to fully comprehend the impact of this technology. Long-term studies examining the impact of CGM on inflammatory processes and vascular health are required to facilitate its integration into diabetes care protocols. As technology advances, continuous glucose monitoring (CGM) offers the potential to improve the quality of life for men with type 1 diabetes mellitus (T1DM) by mitigating ED and other diabetes-related complications while also providing better glycemic control.

## Data Availability

No new data were created in this paper.
